# 3D Anatomy of the Quail Lumbosacral Spinal Canal—Implications for Putative Mechanosensory Function

**DOI:** 10.1093/iob/obaa037

**Published:** 2020-10-30

**Authors:** Viktoriia Kamska, Monica Daley, Alexander Badri-Spröwitz

**Affiliations:** 1 Dynamic Locomotion Group, Max Planck Institute for Intelligent Systems, 70569 Stuttgart, Germany; 2 Department of Ecology and Evolutionary Biology, University of California Irvine, Irvine, CA, USA

## Abstract

Birds are diverse and agile vertebrates capable of aerial, terrestrial, aquatic, and arboreal locomotion. Evidence suggests that birds possess a novel balance sensing organ in the lumbosacral spinal canal, a structure referred to as the “lumbosacral organ” (LSO), which may contribute to their locomotor agility and evolutionary success. The mechanosensing mechanism of this organ remains unclear. Here we quantify the 3D anatomy of the lumbosacral region of the common quail, focusing on establishing the geometric and biomechanical properties relevant to potential mechanosensing functions. We combine digital and classic dissection to create a 3D anatomical model of the quail LSO and estimate the capacity for displacement and deformation of the soft tissues. We observe a hammock-like network of denticulate ligaments supporting the lumbosacral spinal cord, with a close association between the accessory lobes and ligamentous intersections. The relatively dense glycogen body has the potential to apply loads sufficient to pre-stress denticulate ligaments, enabling external accelerations to excite tuned oscillations in the LSO soft tissue, leading to strain-based mechanosensing in the accessory lobe neurons. Considering these anatomical features together, the structure of the LSO is reminiscent of a mass-spring-based accelerometer.

## Introduction

Birds are diverse vertebrates with exceptional ecological range, occupying many habitats on Earth. Birds are capable of aerial, terrestrial, aquatic, and arboreal (tree climbing) locomotion, and many species regularly use more than one locomotor mode. Avian species show specializations in limb proportions, foot morphology, and body shape associated with their specific locomotor ecology and habitat (Grant 1968; Norberg 1979; Carrascal et al. 1990; Gatesy and Middleton 1997; Barbosa and Moreno 1999; Zeffer et al. 2003; Stoessel et al. 2013; Heers and Dial 2015; Daley and Birn-Jeffery 2018). However, despite the diversity in body size and limb proportions (Gatesy and Middleton 1997; Heers and Dial 2015), birds share a consistent body plan, with a similar basic arrangement of bones and joints. This suggests that it is not innovations in body form that has been critical to the exceptional agility and locomotor diversity of birds.

**Fig. 1:**
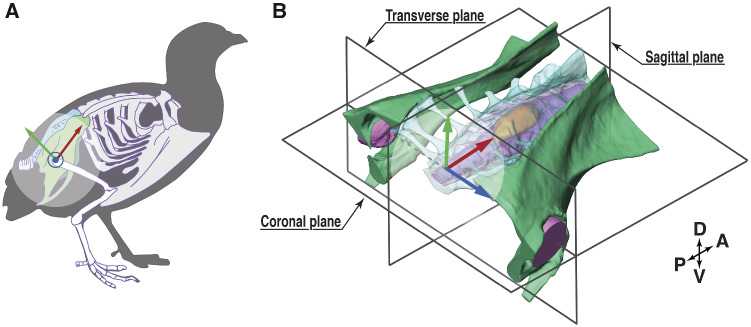
The anatomy of the synsacrum of the quail. (**A**) Schematic skeletal outline of a quail synsacrum, emphasized in green and turquoise. The schematic was modified from Modesto (2009), CC license. (**B**) A 3D view of the synsacrum, also showing a local coordinate system (x-red, y-green, and z-blue, used in “Digital dissection, uCT scanning” section), and three planes of reference. The coordinate origin is located at the center between both femur head sockets, with the coronal plane in-parallel to the orientation of the denticulate ligament network (Fig. 2). Abbreviations are explained in Appendix Table A3.

Birds may possess a novel balance sensing organ in the lumbosacral spinal canal; a structure referred to as the “lumbosacral organ” (LSO), which may contribute to their locomotor agility and evolutionary success (Necker 1999, 2005; Necker 2006). While many tetrapods have enlargements in the cervical and lumbosacral spinal regions to support the sensorimotor demands of pectoral and pelvic limb function—the avian lumbosacral spinal canal is unique among vertebrates (Giffin 1990, 1995). Birds exhibit particularly pronounced enlargement of the lumbosacral canal compared to other tetrapod species (Giffin 1990, 1995; Badawi et al. 1994; Haziroglu et al. 2001). The synsacrum of birds is a holistic structure formed by the fusion of lumbar and sacral vertebrae with each other and with the pelvic girdle (Fig. 1; Baumel 1993). Within the avian synsacrum, the fused lumbosacral vertebrae form an enclosed and enlarged space, substantially larger than the spinal neural tissues contained within. Along with this unique bony morphology, the avian lumbosacral spinal region contains a suite of novel soft tissue morphological features (Sansone 1977; Uehara and Ueshima 1984; Necker 2005). The combined features of the LSO appear to be shared by all birds and are unique to birds among living vertebrates.

**Fig. 2:**
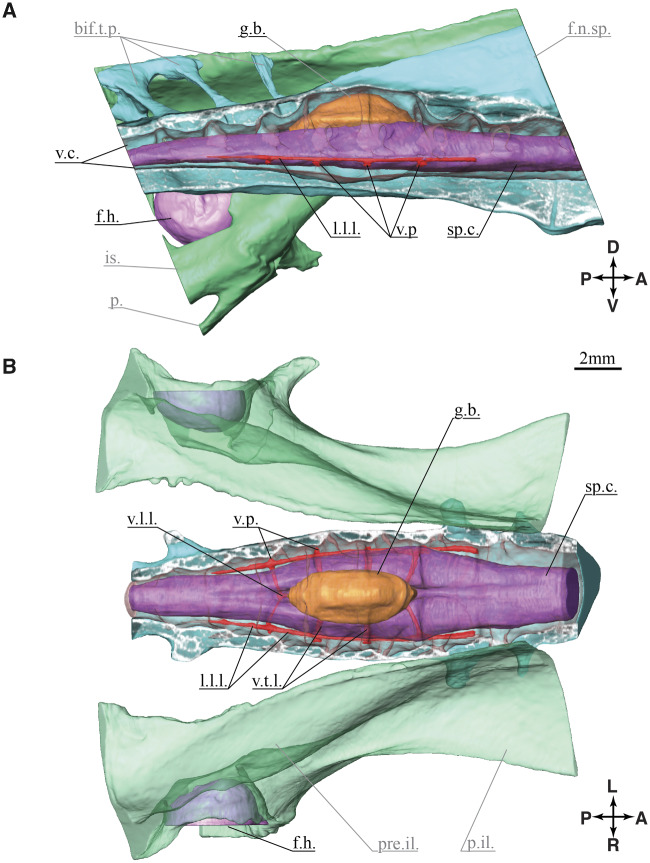
Anatomy of the lumbosacral enlargement. The 3D model is created from the digital dissection. (**A**) Right lateral view at the sagittal plane section of the lumbosacral spine. (**B**) Coronal section view, the canal is cut at half height, soft tissues are not cut.

The LSO structure (Fig. 2) includes segmental bilateral protrusions of neural tissue along the margins of the spinal cord, the accessory lobes of Lachi (Lachi 1889 as cited in Kölliker 1902). These lobes occur near the intersections of denticulate ligaments, a network of ligaments that support the spinal cord (Streeter 1904; Schroeder and Murray 1987; Necker 2005; Necker 2006). The spinal cord is dorsally bifurcated in this region and supports a glycogen body centered and dorsally. The glycogen body is a gelatinous ellipsoid (Fig. 2) composed of highly branched glucose polymers and cells of glial origin (Koizumi 1974; De Gennaro 1982; Benzo and De Gennaro 1983; Necker 2005; Imagawa et al. 2006). The fused lumbosacral vertebrae form a structure with distinctive, segmentally arranged, transverse semi-circular grooves (TGs; or “semi-circular canals” in Necker 1999). The arrangement of TG and accessory lobes is reminiscent of the mammalian vestibular system (Necker 2005; Necker 2006). Each lobe may contain mechanoreceptors (Schroeder and Murray 1987; Necker 2006; Yamanaka et al. 2008) with axons projecting to last-order premotor interneurons in the spinal pattern generating network (Eide 1996; Eide and Glover 1996; Necker 1997; Necker 1999; Rosenberg and Necker 2002). Collectively, these features suggest that the LSO functions as a balance sensing organ involved in the regulation of hindlimb sensorimotor control, independent of vestibular balance sensing in the head.

Although the anatomical features of the avian LSO have been known for decades, the putative mechanosensing function remains controversial and unresolved (Lachi 1889; De Gennaro 1982; Schroeder and Murray 1987; Kölliker 1902; Streeter 1904; Baumel 1993; Rosenberg and Necker 2002). Historically, several hypotheses have been proposed for LSO function, including nutrition storage (Terni 1924; Watterson 1949; De Gennaro 1961; Azcoitia et al. 1985) or a second brain (Streeter 1904). More recently, mechanosensing is the prevailing hypothesis, but at least two theories exist on the nature of the mechanosensing process. Schroeder proposed a strain mechanosensing function, based on the location of the accessory lobes near the intersections of the denticulate ligaments, which could enable stimulation in response to ligament strain (Schroeder and Murray 1987). Alternatively, Necker proposed a balance sensing function of the LSO based on the canal-like shapes within the bone and functional analogy to the mammalian inner ear (Necker 1999, 2005). Necker suggests that cerebrospinal fluid (CSF) flow stimulates mechanosensors in the accessory lobes, enabling the sensation of body movement (Necker 1999, 2005; Necker 2006).

Here we investigate the 3D anatomy of the quail LSO, with a focus on quantifying the geometric and biomechanical properties relevant to a potential mechanosensing function. Necker’s theory for balance sensing requires CSF flow to act upon the accessory lobes in response to body motion. Although it is feasible that CSF flow elicits a mechanosensory response in the accessory lobes, this proposed sensing mechanism does not explain several anatomical features of the LSO. We suggest an alternative mechanosensing hypothesis based on the anatomical features and Schroeder’s observations (Schroeder and Murray 1987)—the inertial load of the glycogen body may act to tune and amplify localized oscillations of the spinal cord neural tissues, resulting in stress and strain in the denticulate ligaments which stimulate the accessory lobes to sense body motion (De Gennaro 1982; De Gennaro and Benzo 1987; Polak-Krasna 2019). Here, we aim to quantify the morphology of the lumbosacral region of the common quail (*Coturnix coturnix*) to establish whether the topological and biomechanical features are more consistent with a fluid-flow or tendon-strain-based mechanosensing function.

No tools are currently available to directly visualize the *in vivo* movements of neural soft-tissues in the lumbosacral region of birds. The region is inaccessible to direct instrumentation because it is fully enclosed in bone, and access would damage the tissues of interest and impair the mechanosensing function (Necker et al. 2000). The region is also difficult to image *in vivo* because of the broad range of contrasts between bones, neural tissue, pneumatized bone, and CSF, which each contribute to the hypothesized mechanosensing functions. For these reasons, we focus on establishing the geometric and tissue properties relevant to developing mechanical models of the mechanosensing function. We use a combined approach of digital dissection of a specimen using diffusible iodine-based contrast-enhanced computed tomography (CT; Metscher 2009; Gignac and Kley 2014), complemented with a classic dissection via a stereo microscope to provide additional detail on the structure and potential deformability of the denticulate ligaments network. We overlay the measurements from classical and digital dissections to provide a complete 3D topology of the common quail LSO.

## Materials and methods

Three specimens were prepared for the classical dissection, and one specimen was prepared for the digital dissection (micro-CT and 3D segmentation). For the digital dissection, one quail specimen was obtained from a commercial breeder (Fayre Game farm, UK) and prepared using fixation and staining procedures recommended by Metscher (2009). The synsacrum was isolated and fixed for 3 days in 10% neutral-buffered formalin. The synsacrum was then immersed in 1% Lugol’s iodine solution (Sigma, Life science, L6146). The specimen was test-scanned to check contrast levels periodically, and a final scan was taken after incubation in Lugol’s iodine for 49 days.

For the classical dissection, three adult, female common quail (*C. coturnix*, Reese and Reese 1962) were obtained as frozen carcasses from a commercial breeder (Kitzingen, Germany). The birds’ average weight was 170 ±  5 g, the body size varied between 10 and 12 cm. All carcasses were dissected according to the practice described in Gurtovoy et al. (1992). After slow thawing, the synsacrums were isolated and stored in a closed vessel at 4°C temperature to minimize dehydration and soft-tissue autolysis.

### Digital dissection, uCT scanning

The quail specimen was scanned with a micro-CT machine (Skyscan 1172), operated at 80 kV and 124 uA. About 1030 raw images (dark object against a bright background) were taken within one 360° rotation, with an increment of 0.35°, and an integration time of 500 ms per image. We averaged three images at each angle position. The raw images are 1000 × 668 pixels in size. Voxels of the original image stack are isotropic, with 27 μm edge length. The images were back-projected to generate 634 images (TIFF format, bright signal against a black background). The resulting image size is 1000 × 1000 pixels.

### Segmenting uCT data

The raw data were 3D segmented in Amira version 6.5.0 (Visage Imaging). The back-projected image stack was directly used in Amira and required no further aligning. The contrast from dense bone tissue allowed labeling with a threshold function. The soft tissue inside the lumbosacral vertebral canal did not have enough contrast for the threshold function. Instead, soft tissues were segmented manually, with the “paintbrush,” the “magic wand,” and the “lasso” tool. We switched working planes when necessary, depending on the shape and orientation of anatomical structures. The segmented structures were interpolated, from two to seven labeled slices.

### 3D segmentation

Surfaces were individually rendered with the module “Surface Gen.” “Label Field” files (Amira Mesh [AM] format) were split, one for each segmented structure (Ruthensteiner and Heß 2008). For rapid processing, 3D model surfaces were closed and the number of triangles reduced (Molnar 2012). 3D segment surfaces were exported (Standard Triangle Language [STL] format) and loaded in Autodesk Maya. Several of the resulting structures overlapped by a few triangles and formed nonmanifold edges. These overlaps were manually treated by removing nonmanifold edges and reducing the number of polygons. Any remaining holes were manually closed and smoothed with polygonal meshes with the “Sculpt Geometry” tool (Buchanan et al. 2014). The final meshes were exported (STL format) and re-imported into Amira for the geometric morphometric analysis.

### Digital dissection of denticulate ligaments

**Appendix Fig. A1:**
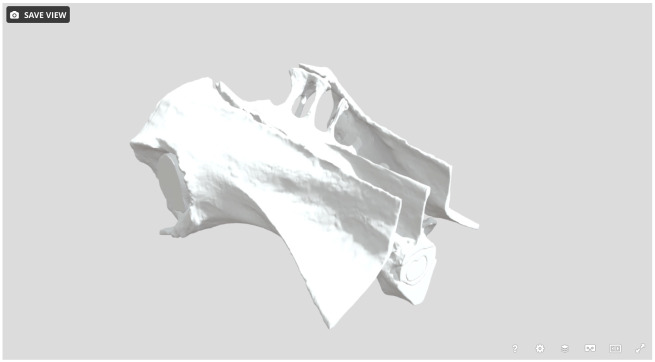
Snapshot of the 3D model of the lumbosacral region. The model contains eight objects, and is shared via SketchFab https://skfb.ly/6U6Ws.

The denticulate ligaments are the smallest anatomical structures we were able to resolve with the digital dissection. The ligaments barely appeared in the pre-sliced raw data, with a thickness of a few voxels (27 μm voxel size). With a denticulate ligament thickness close to the imaging resolution, they required a dedicated segmentation. The first, main slicing direction was chosen approximately along the spinal canal direction, and only a few slices contained recognizable denticulate ligaments. After this first segmentation of the spinal canal, the glycogen body, and the spinal cord, we re-sliced the raw data in-parallel to the denticulate ligaments’ expected orientation and imported the new slices into Amira, which largely improved denticulate ligament visibility. This new set was used to segment the denticulate ligaments with the “paintbrush” tool. Both sets were merged by overlaying the commonly visible ventral processes (Fig. 2B). The supplementary video displays the reconstructed morphologies; the glycogen body, spinal cord hemispheres, and the denticulate ligaments within the spinal canal filled with cerebrospinal fluid. A 3D model of the reconstruction is available in Appendix Fig. A1.

### Classical dissection

**Fig. 3:**
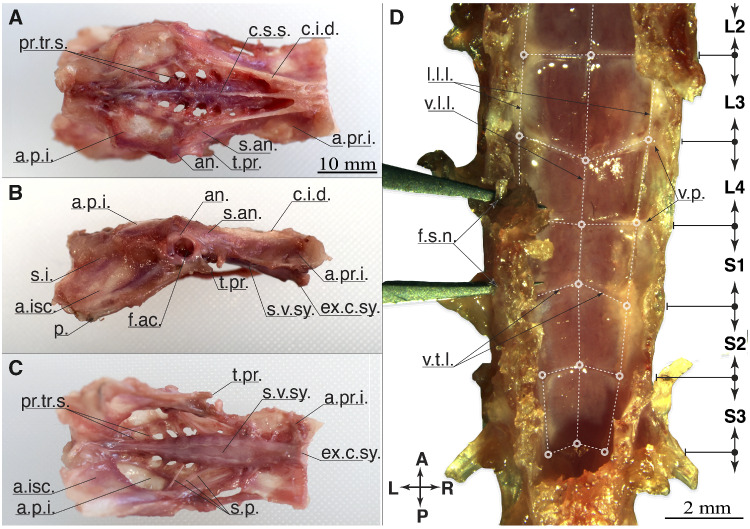
Anatomy of the synsacrum, classic dissection. (**A**) dorsal, (**B**) right lateral, and (**C**) ventral view. (**A–**C) shows the intact synsacrum with a closed lumbosacral canal. Posterior left and anterior right (**D**) show the spinal canal opened and from dorsal. Glycogen body and spinal cord are removed, to reveal the denticulate ligaments in the region of the glycogen body (S2–L4). The denticulate ligaments are emphasized by dashed white lines, and the network’s nodes by circles. The tips of a pair of tweezer are inserted through the foramina of the S1 and L4 vertebrae.

With the classical dissection, we exposed the fine structure of the denticulate ligaments. Synsacrum bone surfaces were cleaned of flesh. The movable thoracic and caudal vertebrae were removed. The synsacrum surfaces were photographed (Nikon D5500, lens Nikon AF-S Nikkor 35 mm f/1.8G ED). Three perspectives are shown in Fig. 3A–C. The remaining classical dissection was performed under a fluorescent stereomicroscope (Leica M205 Fa, magnification 7.8–160×). Images from the microscope dissection were taken with the built-in camera (Leica DFC digital 7000-T, 2.8 megapixel sensor, pixel size 4.54 μm). Related pictures in Figs. 3D, 9, and 8B have been cropped and annotated for clarity and were not otherwise altered.

To simplify the classical dissection the bones of the pelvic girdle were removed. The spinal canal wall was opened from the dorsal side, along the coronal plane aligned to the intervertebral foramina location. The spinal cord and the glycogen body were removed from the dorsal side of the spinal canal. The denticulate ligaments were photographed (Fig. 3D), and ligaments were manipulated with a pair of forceps to indicate their stretchability and connectivity (Fig. 9).

### Measurements

**Fig. 4:**
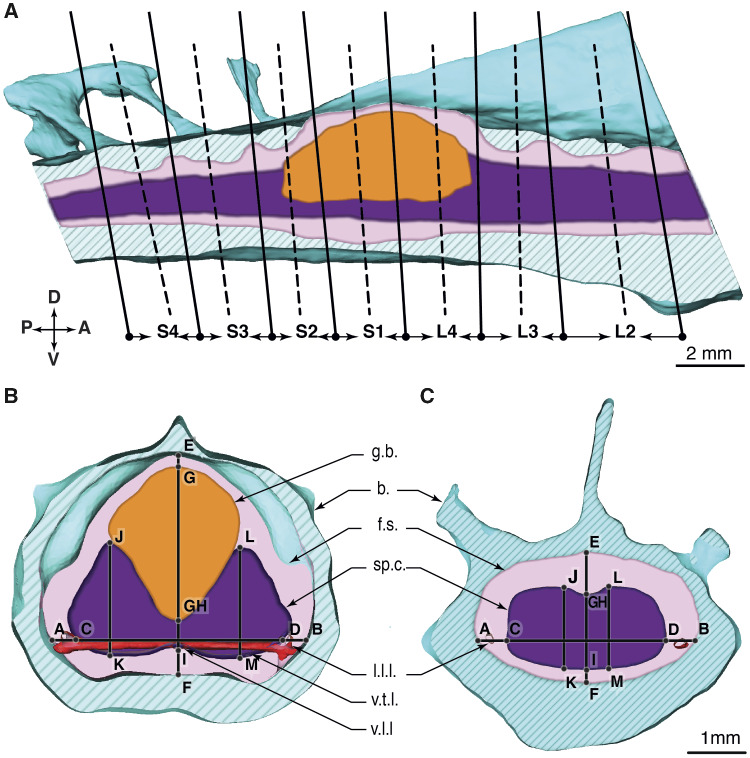
The lumbosacral vertebral sections of a quail, schematic view. (**A**) Sagittal section through the spinal canal, right lateral view. Solid black curves present the cut planes at the vertebral fusion regions. Dashed black curves are the cut planes at the vertebral middle regions. (**B**) Transverse section through the vertebral column at the fusion region of the S1 and L4. The glycogen body spans between vertebrae S2–L4. (**C**) Transverse section between L3 and L2.

Dimensions and distances between anatomical structures were measured by applying landmarks to the 3D model in Amira (Fig. 4). We measured in the transverse cut planes (Fig. 4B), with a slight angle adjustment (Fig. 4A). The cut planes were placed at the vertebral fusions (dashed lines, Fig. 4A) and mid-vertebral regions (continuous lines). The glycogen body spans from the fourth lumbar to the second sacral vertebra (L4, S1, and S2). We show a cut through the spinal canal in regions with and without the glycogen body visible in Fig. 4B, C, respectively.

### Volume measurements

Partial and full volumes of the spinal cord, the glycogen body, and the CSF were extracted in Autodesk 3ds Max. We used the transverse cut planes to separate vertebrae, as shown in Fig. 7A–C. At the position of the cut planes, the structures were split with the “Slice modifier” function. Slicing volumes creates openings in the polygonal mesh. We closed these with the options “Cap holes” function and “Smooth new faces.”

### Ligament strain calculation

**Fig. 5:**
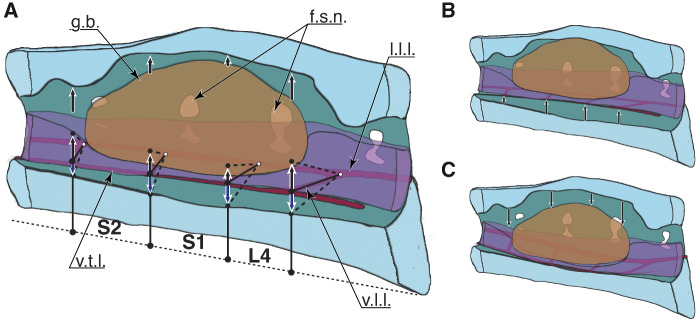
Potential for soft tissue movement and the resulting ligament deformation, schematic illustration. (**A**) Soft tissue in the (**A**) resting, (**B**) upper, and (**C**) lower position. Red indicates the transverse ligaments. Black arrows show up- and down-movements of the glycogen body, the spinal cord tissue, and the ligament nodes. The nodes are defined as the intersection of the ventral transverse and ventral longitudinal ligaments. The dashed lines show the ventral transverse ligaments when deformed.

To explore the potential for soft tissue oscillations caused by external accelerations that could drive a mechanosensing process, we estimate the maximum possible displacement within the spinal canal, based on the available space. The resulting deformations would include all soft tissues, and the ligament network would produce countering forces, similar to a hammock stretched when loaded by a person (Fig. 5). For simplicity, we assume that the soft tissue moves in parallel to the sagittal plane, driven by in-plane accelerations of the synsacrum.

**Fig. 6:**
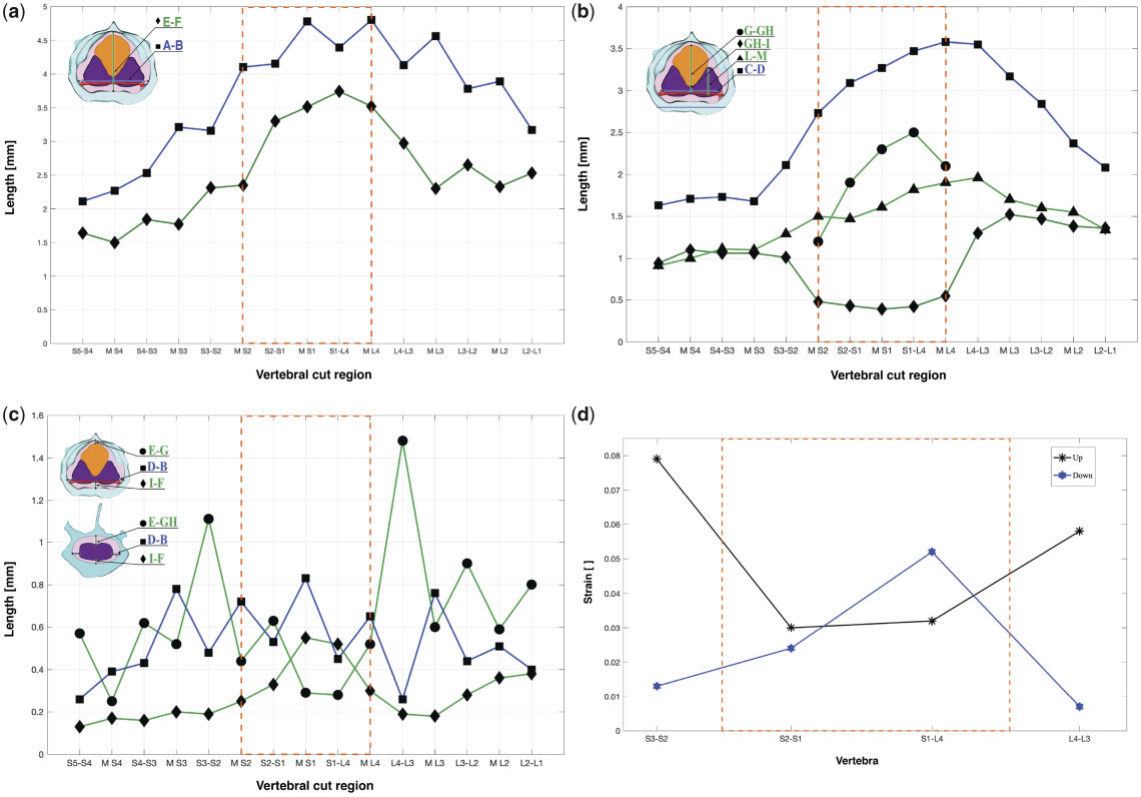
(**a**) Height and width of the spinal canal by spinal segment. **A-B** (blue curve, square markers): spinal canal width. **E-F** (green curve, diamond markers): spinal canal height. (**b**) Morphometrics of the neural soft tissue. **C-D** spinal cord width with square markers, **G-GH** glycogen body height with circle markers; **GH-I** middle of the spinal cord height shown with triangle markers, **L-M** spinal cord hemisphere height, diamond markers. (**c**) CSF space morphometry. **E-G** vertical distance between the glycogen body top and the spinal canal dorsal (circle markers), **E-GH** vertical distance between the spinal cord and the spinal canal dorsal in the range outside the glycogen body (circle markers), **I-F** vertical distance between the spinal cord ventral surface and the spinal canal (diamond markers), **D-B** horizontal, lateral distance between spinal cord and the spinal canal (square markers). (**d**) Strain estimates of the transverse ligaments at their maximum ventral and dorsal positions of the spinal cord, in reference to their resting position. Asterisk markers (black line) show the maximal transverse ligament strain at the dorsal position, and hexagram markers (blue line) indicate maximal strain in the ventral position.

We estimate the transversal ligament strain with a simplified model. The initial state assumes unloaded and nonstretched ligaments in planar orientation. We measured lengths (L) of left and right transverse ligaments of the three classical dissection specimens and one digital dissection specimen and averaged values per vertebra (Table 3). The fluid space below and above, extracted from the digital dissection (Fig. 6c), provided the vertical range, with additional heuristics—we averaged two neighboring mid-vertebra values for the upward (dorsal) fluid distance estimate (Fig. 6c, E-G and E-GH, i.e., the average of M-S3 and M-S2 for S3–S2). Otherwise, we would overestimate the potential displacement based on the additional height of TGs between vertebrae. For downward (ventral) distance estimate, we directly used ventral distance in the CSF space below the spinal cord (Fig. 6c, I-F). We then calculated the maximally stretched length at maximum vertical displacement of a transverse ligament using the Pythagorean theorem and its nominal strain $e=\frac{l-L}{L}$.

**Table 3:** The calculated strain of a transverse ligament caused by virtual, vertical soft tissue oscillations

Vertebral range	S3–S2	S2–S1	S1–L4	L4–L3
Dorsal fluid space (up) (mm)	0.48	0.37	0.41	0.56
Ventral fluid space (down) (mm)	0.19	0.33	0.52	0.19
Transverse ligament length $\bar{L}$, at rest (mm)	1.21	1.51	1.61	1.67
Transverse ligament length $\bar{l}$_up, virt_ stretched (mm)	1.30	1.55	1.66	1.77
Transverse ligament length $\bar{l}$_down, virt_ stretched (mm)	1.23	1.54	1.69	1.68
Nominal strain $\bar{e}$_up_ (%)	7.9	3.0	3.2	5.8
Nominal strain down $\bar{e}$_down_ (%)	1.3	2.4	5.2	0.7

**Appendix Table A1:** The length in (mm) of each vertebra and the glycogen body

Morphology	S4	S3	S2	S1	L4	L3	L2	GB
Length x-direction (mm)	2.2	2.3	1.9	2.2	2.4	2.2	3.1	5.2

Lengths were measured from the digital model, at the y-height of the denticulate ligament network.

**Appendix Table A2:** Ligament intersection angles, numbers are mean (SD) angles over two or three classical dissection specimen

S3–S2	S2–S1	S1–L4	L4–L3	L3–L2
72(5)°	71(6)°	84(4)°	98(7)°	92(10)°

**Appendix Table A3:** Scientific abbreviations and symbols

Abbreviation	Name	Abbreviation	Name
A	Anterior	a.isc.	Ala ischii
b.	Bone	a.p.i.	Ala postacetabularis ilii
COM	Center of mass	a.pr.i.	Ala preacetabularis ilii
CSF	Cerebrospinal fluid	an.	Antitrochanter
f.ac.	Foramen acetabuli	bif.t.p.	Bifurcated transverse processes
f.h.	Femural head	c.i.d.	Crista iliaca dorsalis
f.s.n.	Foramina spinal nerve	c.s.s.	Crista spinosa synsacri
g.b.	Glycogen body	ex.c.sy.	Extremitas cranialis synsacri
l.l.l.	Lateral longitudinal ligament	f.n.sp.	Fused neural spines
P	Posterior	i.	Ilium
sp.c.	Spinal cord	is.	Ischium
TG	Transverse semi-circular grooves	p.	Pubis
v.c.	Spinal canal	p.il.	Postacetabular ilium
v.l.l.	Ventral longitudinal ligament	pr.tr.s.	Processus transversus sacralis
v.p.	Ventral process	pre.il.	Preacetabular ilium
v.t.l.	Transverse ligament	s.an.	Sulcus antitrochantericus
		s.i.	Sync. ilioischiadica
		s.v.sy.	Sulcusvent. synsacri
		t.pr.	Tuberculum preacetabulare

### Effective force from submerged, high-density glycogen body

All LS tissues are submerged in CSF, which itself has a specific gravity $\mathrm{SG}=\frac{\rho (\mathrm{CSF})}{\rho (\mathrm{water})}=1.00,$ whereas the specific gravity of spinal cord tissue indicates a small tendency for sinking (Table 1). In comparison, glycogen is considerably denser, which suggests the glycogen body substantially loads the spinal cord and denticulate ligaments with a normal force $F_{N}$ corresponding to the glycogen body’s effective, submerged weight. The estimated force value can assist future research characterizing the LS structure during external accelerations, *in silico*, or in physical experimental setups. It presents the intrinsic force applied to the LS structure. For a future calculation of soft tissue deformation from force loading, more data will be required, including the externally applied forces caused by acceleration, the materials’ elastic, anisotropic properties, and the boundary conditions restraining the spinal cord on its anterior and posterior ends.

**Table 1:** Densities *ρ* and specific gravity *ρ*/*ρ*H20 of soft tissue inside the LS spinal canal

Details	Density *ρ* [g/cm^3^]	Specific gravity [ ]	References
CSF	1.00	1.00	Higuchi et al. (2004); Lui et al. (1998) (human)
Spinal cord	**1.04**	1.04	Siegal (1988) (rat spinal cord)
Glycogen	1.40, …, 1.48	1.40	Scott and Still (1970; rat liver, human leukocytes)

Values marked in bold are calculated based on data provided in the referenced literature. We apply the glycogen density’s lower bound in our calculations.

Here, we calculate the effective force applied by the glycogen body onto the spinal cord and the spinal cord at the denticulate ligament, and assume a simplified system (1) at atmospheric pressure, (2) without pressure from the height of a fluid column, and (3) with incompressible soft tissues.

The extracted soft tissue volumes are provided in Table 2. The effective, normal force $F_{N,\mathrm{eff}}$ of tissue submerged in CSF is calculated as:

(1) $$\begin{array}{c} F_{N,\text{eff}}=m_{\text{ti}}\cdot g-\rho_{\text{CSF}}\cdot V_{\text{ti}}\cdot g\\ =\rho_{\text{ti}}\cdot V_{\text{ti}}\cdot g-\rho_{\text{CSF}}\cdot V_{\text{ti}}\cdot g\\ =\Delta \rho \cdot V_{\text{ti}}\cdot g \end{array}$$

where $m_{\mathrm{ti}}$, $V_{\mathrm{ti}}$, and $\rho_{\mathrm{ti}}$ are the mass, volume, and density of the tissue of interest, respectively. $\rho_{\mathrm{CSF}}$ is the density of CSF, and $g\;=\;9.81\;m/s^{2}$ is the gravitational acceleration. The glycogen body’s specific gravity ($SG_{\mathrm{GB}}\;=\;\frac{\rho_{\mathrm{GB}}}{\rho_{\mathrm{CSF}}}$) provides a similar understanding of its floating vs. sinking behavior when submerged in CSF.

**Table 2:** Digitally measured soft tissue volumes in [mm^3^] and modeled effective normal forces (F_N_ in [μN]) of the glycogen body, in order of vertebrae

Vertebra	Measure [unit]	S4	S3	S2	S1	L4	L3	L2	∑(S4, …, L2)
Spinal cord	Volume [mm^3^]	2.8	3.9	5.5	7.5	11.8	10.7	10.2	52.4
Glycogen body	Volume [mm^3^]	–	–	1.4	6.8	4.8	–	–	13.0
CSF	Volume [mm^3^]	3.3	5.6	8.3	11.5	10.8	14.3	10.8	64.6
Spinal canal/∑	Volume [mm^3^]	6.1	9.5	15.2	25.8	27.4	25.0	21.0	130.0
Glycogen body	F_(N, eff , 1.40)_[μN]	–	–	5.5	26.7	18.8	–	–	51.0

### Nomenclature

We assigned the intersection between lumbar and sacral vertebra numbering according to the maximum extension of the glycogen body and the spinal canal (Catala et al. 1995; Can and Özdemir 2011). We also matched the denticulate ligament networks from the classical dissection and the digital dissection by overlaying them. Similar nomenclature is used by Necker (2005) for pigeons.

## Results

We characterize the geometry (length, surface area, and volume) and the topology of the lumbosacral enlargement and its soft tissues. We report the morphometric data of the spinal cord hemispheres, the glycogen body, the denticulate ligaments, the CSF, and the spinal canal, for use in the development of physical models of the mechanosensing process. Based on the higher density of the glycogen body compared to the surrounding CSF, we calculate an estimate of its normal force applied to the spinal cord and the denticulate ligaments. We also estimate the maximum likely ligament strains from deforming the denticulate ligaments.

### Morphometrics

The spinal canal data between vertebrae L2-S4 shows the following trends: (1) The overall height and width of the canal reduce from anterior to posterior, from 2.5  and 3.2 mm (H/W at L2–L1) to 1.7  and 2.2 mm in S5–S4, respectively (Fig. 6a). (2) The glycogen body is co-located in the region of a substantial spinal canal extension in all directions, between the three vertebrae L4, S1, and S2. (3) Fused vertebrae are connected by TGs, which substantially alter the canal shape. At these TGs, we observe maxima in the dorsal vertical direction (Fig. 6a, diamond markers), with the largest vertical extension at S1–L4 (3.7 mm). Unlike the vertical extension, lateral extension maxima are found in the middle of the vertebrae (Fig. 6a). The S1 and the L4 vertebrae contain the largest lateral extension (MS1, ML4, 4.8 mm). Figure 6a shows that the spinal canal width is always greater than its height in the lumbosacral region. The glycogen body is 5.2 mm long (X-direction, Appendix Table A1) and has its maximum height of 2.5 mm at the dorsal fusion zone S1–L4, where the spinal canal height reaches a maximum of 3.7 mm (Fig. 6b, G-GH).

We provide two sets of vertical extension data of the spinal cord, a first from the intersection with the sagittal plane and a second from the intersection with the parasagittal plane, at its highest point. We see a drop in the spinal cord’s vertical extension in the sagittal cut plane between L4 and S2, where it leaves an only 0.4 mm high bridge between both hemispheres (Fig. 6b, GH-I). Here both hemispheres remain barely connected by spinal cord material. The vertical spinal cord extension in the parasagittal plane reaches its maximum between L3 and L4, with a height of 1.9 mm (Fig. 6b, GH-I). In sum, we see that the spinal cord width (Fig. 6b, C-D) and height (Fig. 6b, L-M) decrease from anterior to posterior, except in the region of the glycogen body.

The height of the CSF above the spinal cord fluctuates considerably in the range of L1–S4 (Fig. 6c, round markers). TGs are located between dorsal and lateral surfaces inside the spinal canal. The CSF data show TGs as spikes in the curve. The CSF has its highest vertical extension (1.5 mm) at TG L3–L4 (Fig. 6c). The smallest height of CSF (0.3 mm) is found directly above the glycogen body, at S1 and TG S1–L4 (Fig. 6c, E-G).

The vertical extension of CSF below the spinal cord reduces, on average, from anterior to posterior, from 0.4 to 0.1 mm, respectively (Fig. 6c, I-F). A large “dip” is visible for about two vertebrae between S1–L4 and M-S1, where the fluid space below the spinal cord and the denticulate ligaments triples; it expands from 0.2 to 0.6 mm. The maximum ventral expansion coincides with the maximum height of the glycogen body (Fig. 6b, G-GH) and the maximum spinal canal height (Fig. 6a, E-F).

The CSF surrounds the soft tissues in the spinal canal. The lateral expansion of CSF reduces from anterior (2 × 0.4 mm) at L2-L1 toward posterior at S5–S4 (2 ×  0.15 mm). The shape of the fluid space shows a zig-zag pattern, where lateral fluid expansions in the middle of vertebrae coincide with the location of spinal nerve foramina, also because of the height of the cut plane (i.e., MS1, MS2, etc., Fig. 6c, D-B). The remaining data at vertebrae fusions indicate that the foothills of the TGs extend dorsally into the spinal canal walls (i.e., S1–L4, S2–S1, etc.). The most posterior Segment S4 shows no peak lateral expansion. Its smaller foramina are shifted posterior, toward S4–S5.

### Volume measurements

**Fig. 7:**
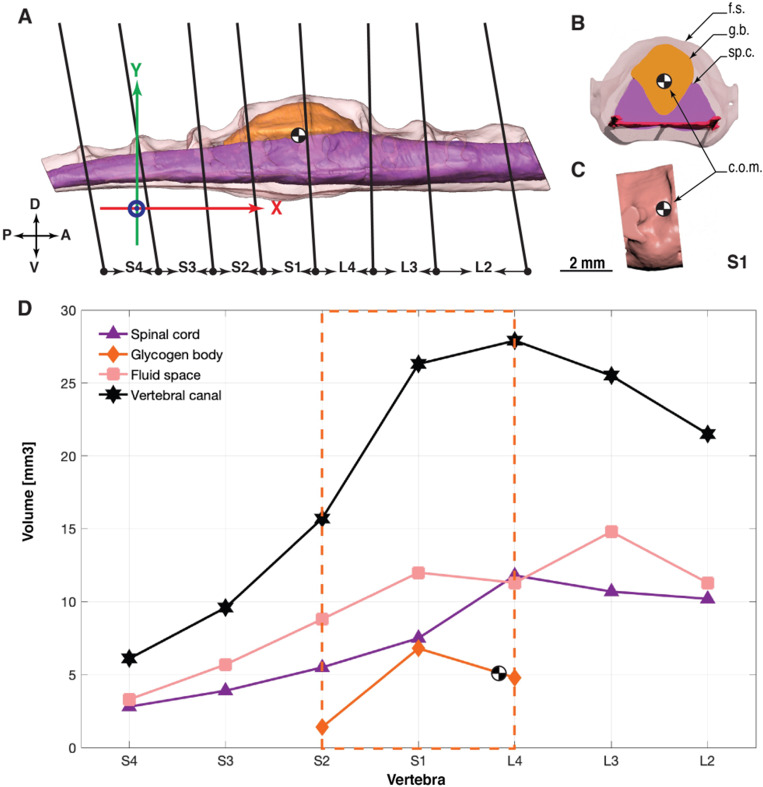
Volume measurements by spinal canal segments. (**A**) Right lateral view at the 3D model of the spinal cord, the glycogen body, and the CSF surrounding both. Cuts are placed along the anatomical fusion zone, hence they are slightly tilted. The coordinate system’s origin is placed between the geometric center of both femur head sockets, with the *x*-axis aligned in-parallel with the denticulate ligament. The horizontal distance (x-axis) between the glycogen body’s center of mass and the femur head (coordinate system center) is 6.2 mm, the radial distance is 6.8 mm. (**B**) Segment S1 of the spinal canal, and its contents. (**C**) Right lateral view at the isolated Segment S1 of the spinal canal. (**D**) Data points show the volume measurements per spinal canal segment.

The uCT scan ranges over seven vertebrae, from L2 to S4 (Fig. 7A). An overview of the volume data is provided in Table 2 and Fig. 7D. From L2 to L4, the spinal canal volume per segment increases from 21.0 to $27.4\;mm^{3}$, respectively. The following canal segments then continuously decrease in volume, from S1 with $25.8\;mm^{3}$ to S4 with $6.1\;mm^{3}$ (Table 2 and Fig. 7). The spinal cord volume decreases on average from anterior to posterior, from $10.2\;mm^{3}$ at L2 to $2.8\;mm^{3}$ at S4; however, an maximum in spinal cord tissue occurs at L4, with a volume of $11.8\;mm^{3}$. In total, 50% of the lumbosacral spinal canal is filled with CSF ($64.6\;mm^{3}$ of $130\;mm^{3}$). The spinal canal segments with the glycogen body contain relatively less liquid, with $39\%\;(10.8\;mm^{3})$ of L4 fluid-filled, and 45% of the S1. At spinal Segment L4, the CSF occupies nearly the same volume as the spinal cord: 10.8 and $11.8\;{\mathrm{mm}}^{3}$, respectively.

Most of the glycogen body’s volume is located in S1, with a volume of $6.8\;mm^{3}$, or 52% of its total volume of $13{\;\mathrm{mm}}^{3}$ (Table 2 and Fig. 7). An additional 37% ($4.8{\;\mathrm{mm}}^{3}$) of the remaining glycogen body is located in L4 and 11% ($1.4\;{\mathrm{mm}}^{3}$) is located in S2. Within the L4-S2 segments, the glycogen body occupies 19% spinal canal volume and has half the volume of the sum of local spinal cord segments ($13.0\;mm^{3}$ vs. $24.8\;mm^{3}$, 52%).

### Normal forces by submerged glycogen body

Equation (1) indicates that forces applied by the dorsally positioned glycogen body depend linearly on the size and density of the glycogen body (Tables 1 and 2), leading to a net normal force of $F_{N,\mathrm{eff}}\;=\;13\cdot 10^{-9}m^{3}\cdot 0.40\cdot 10^{3}\frac{\mathrm{kg}}{m^{3}}\cdot 9.81\frac{m}{s^{2}}\;=\;51\cdot 10^{-6}N\;=\;51\;\mu N$ applied by the glycogen body, at the ventrally located spinal cord and the denticulate ligaments (Table 2). In comparison, the higher-than-CSF-specific gravity (SG = 1.04) of spinal cord sections between S2, S1, and L4 $\left(\sum V_{S2,S1,L4}=24.8\cdot 10^{-9}m^{3}\right)$ creates a normal force of $F_{N}{,}_{\mathrm{eff}}\;=\;10\;\mu N$ directed at the denticulate ligaments.

### Denticulate ligament network

**Fig. 8:**
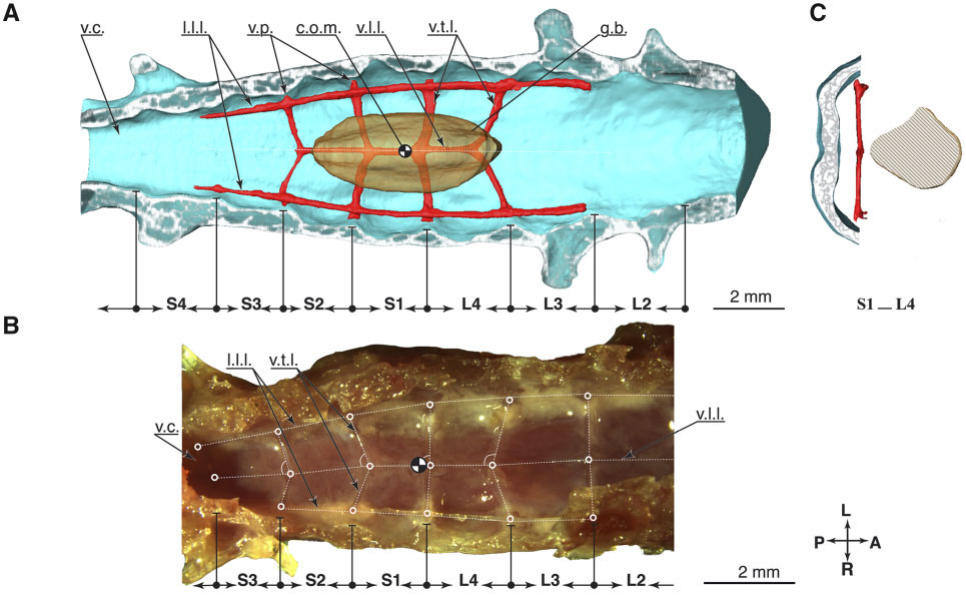
Details denticulate ligaments. (**A**) Digital dissection, top view at the opened spinal canal. (**B**) Classical dissection, else identical to **A**. (**C**) Transverse section through the spinal canal at the S1–L4 fusion region.

We segmented the denticulate ligaments in Fig. 8A from uCT data with a resolution between 1 and 5 voxels (27 μm voxel edge) per ligament diameter. Lateral longitudinal ligaments are thicker, around 3–6 voxels in diameter, and ventral longitudinal ligaments were between 1 and 2 voxels in diameter. We further studied the denticulate ligaments with classical dissection (Fig. 8B). The denticulate ligaments network consists of lateral and ventral longitudinal ligaments (Schroeder and Murray 1987) aligned in a paracoronal plane. Other ligaments intersect in the transverse plane. Ligament nodes present the intersection points.

We measured the intersection angle in reference to the ventral longitudinal ligament (Fig. 8B and Appendix Table A2). Transverse ligaments in the LSO region show changing pennation angles, with angles between 71° (S2–S1) and 90° (L3–L2). The center of mass of the glycogen body projects close to the transverse ligament-pair connecting to the ventral longitudinal ligament at 84° (S1–L4, Fig. 8B).

### Estimating strain denticulate ligaments

**Fig. 9:**
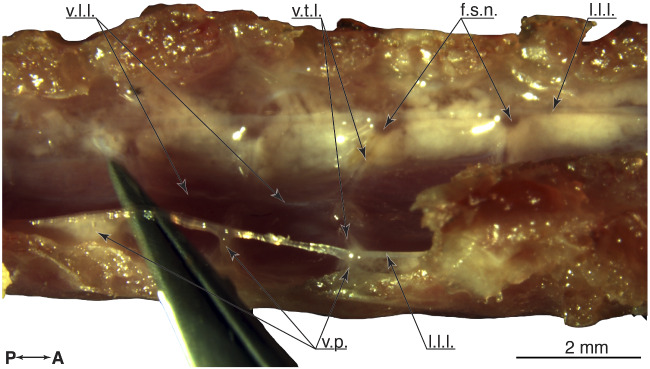
Top view at the open spinal canal, showing the network of denticulate ligaments. The glycogen body and the spinal cord were removed. The ligaments are attached to the spinal canal via ventral process, at the fusion zones between vertebrae. The right lateral longitudinal ligament is raised by a pair of tweezers, and the deformation shows the attachment points with the spinal canal. Anterior is to the right.

Our calculations estimating denticulate ligament strains show that the lowest transverse ligament strain would occur during downward motion in L4–S3 (1%), and the highest strain in S2–S3 during upward movement (8%, Table 3), with an average strain over all transverse ligaments of 5% for upward motion, and 2% for downward motion. During the classical dissection, we manipulated ligaments with a pair of forceps and observed that they are easily stretched to these extents without rupture (Fig. 9).

## Discussion

We generated a 3D anatomical model of the lumbosacral region of the common quail, including the soft tissue and bony spinal canal elements, with a focus on establishing the morphological topology and biomechanical properties relevant to potential mechanosensing functions. This effort aimed to establish quantitative and openly-accessible data on this inaccessible structure for use in physical models and simulations. We observe a hammock-like network of denticulate ligaments supporting the spinal cord in the lumbosacral region, with a close association between the ligament intersections and the known locations of the accessory lobes. The glycogen body likely has higher specific gravity than the surrounding CSF and soft tissues and applies a higher effective normal force compared to the spinal cord in the same area (51 μN vs. 10 μN, respectively), suggesting the potential to pre-stress the denticulate ligament network and allow external accelerations to excite oscillations in the LSO soft tissue. Oscillations in the glycogen body, the spinal cord, and the denticulate ligaments could allow mechanosensing in the accessory lobes neurons. Considering these anatomical features together, the structure of the LSO is reminiscent of an accelerometer.

Many studies have investigated potential functions of the LSO of birds, and the constituent elements, including the glycogen body, the transverse grooves of the bony spinal canal, the accessory lobes, the denticulate ligaments, and the CSF—yet, concrete evidence of LSO function has remained elusive. Early hypotheses considered a nutritional or secretory function of the glycogen body (Benzo and De Gennaro 1981; Azcoitia et al. 1985), or suggested function related to myelin synthesis for the spinal nerves of birds (De Gennaro and Benzo 1976, 1978; Benzo and De Gennaro 1981). Watterson (1954) suggested that the glycogen body plays a vital role in the in-ovo development of avian spinal networks by wedging the spinal cord hemispheres apart during the development (Watterson 1949, 1954). However, none of these hypotheses have yielded conclusive evidence of LSO or glycogen body function.

Most recent work has supported the hypothesis of a mechanoreceptive function of the LSO. In part, this hypothesis is based on the identification of mechanoreceptive neurons within the accessory lobes (Schroeder and Murray 1987; Eide 1996; Eide and Glover 1996; Rosenberg and Necker 2002; Necker 2006; Yamanaka et al. 2012, 2013). Intraspinal mechanosensors are known to exist in many vertebrates (Dale et al. 1987; Wyart 2009; Hubbard et al. 2016; Henderson et al. 2019), including the edge-cells in lampreys, which have been most directly characterized (Rovainen 1979; Grillner et al. 1981; Di Prisco et al. 1990; Hsu et al. 2013). Marginal neurons with apparent developmental homology to lamprey edge cells have been observed in many vertebrates, including reptiles, mammals and amphibians, and the accessory lobes of birds (Grillner et al. 1981; Schroeder and Richardson 1985; Schroeder 1986a, 1986b; Schroeder and Murray 1987; Schroeder and Egar 1990; Hsu et al. 2013). The ultrastructure of the marginal nuclei, including the avian accessory lobes, suggests a close association with denticulate ligaments and morphology similar to peripheral mechanosensors and suggests a strain or pressure sensing function (Schroeder and Richardson 1985; Schroeder 1986a, 1986b; Schroeder and Murray 1987; Schroeder and Egar 1990). These studies suggest the widespread presence of intraspinal mechanosensors among vertebrates.

If the accessory lobes act as intraspinal mechanosensors in birds, the sensory signals will likely integrate directly with the lumbosacral spinal rhythm generation. The homologous edge cells of lamprey integrate the sense of spinal bending directly with the rhythm generating circuits, entraining right/left alternation of activity (Rovainen 1979; Grillner et al. 1981; Di Prisco et al. 1990; Hsu et al. 2013). Similarly, Eide found that chick accessory lobes project onto the contralateral ventral horn near lamina 8 (Eide 1996; Eide and Glover 1996), with potential synaptic targets including last-order premotor interneurons (Eide 1996; Eide and Glover 1996; Necker 2006). This suggests the potential for the accessory lobes to play a direct role in coordinating hindlimb rhythm generation. Additionally, spinal cord lesion studies in chicks revealed that the circuits required for locomotor rhythm generation are completely localized to the ventral spinal cord (Sholomenko and Steeves 1987; Ho and O’Donovan 1993). These lines of evidence suggest direct integration of accessory lobes mechanosensing into the rhythm generating interneuronal networks of the avian lumbosacral spinal cord.

Although the evidence for LSO mechanosensing in birds remains indirect, there is also separate experimental evidence that birds possess balance sensing in the body, independent from the head’s vestibular system. Birds retain the ability to reflexively compensate for body rotations even after labyrinthectomy and spinal cord transection to eliminate descending inputs influenced by vision and vestibular sense (Biederman-Thorson and Thorson 1973). However, the definitive source of this extra-labyrinthine balance sense has remained unresolved.

We observe the following features of the avian LSO and suggest an updated hypothesis for its mechanoreceptive function. The spinal canal around the glycogen body shows a distinct modification of the fluid space and ligament geometry, centered around the glycogen body’s center of mass. The glycogen body has its largest height directly below the S1–L4 fusion zone (Fig. 6b), and the largest ventral CSF space below this region. The denticulate ligament network shows a pennate angular arrangement with an angle close to perpendicular at the S1–L4 fusion (Fig. 8A). The denticulate ligaments extend from the ventral process to attach to the spinal canal walls at the fusion zones between vertebrae (Figs. 9 and 8A, B; Schroeder and Murray 1987). The transverse ligaments directly extend from ventral processes and intersect with ventral longitudinal ligaments at varying pennate angles oriented “towards” the S1–L4 fusion zone (Fig. 8A, B). Ventral longitudinal ligaments and transverse ligaments were the thinnest ligaments observed, with a diameter between 27 and 54μm, compared to the larger lateral longitudinal ligaments with diameters 120–180 μm (Fig. 9). The morphology suggests a varying and anisotropic stiffness of the denticulate ligament network, with a topology resembling a hammock centered directly underneath the glycogen body.

In most regions of the spinal column, spinal cord tissue is held relatively immobile by denticulate ligaments, membranes, the spinal canal itself, and the surrounding CSF (Schroeder and Murray 1987; Loth et al. 2001; Necker 2005; Polak et al. 2014). CSF’s buoyancy supports the spinal cord and protects it from injuries (Polak-Krasna 2019). However, within the avian LSO, the lumbosacral canal structure is modified in ways that are likely to enhance dorsoventral spinal cord motion, by the added mass of the glycogen body, the arrangement of the denticulate ligament network, and the increased dorsal and ventral fluid space within the bony canal.

The physical structure of the LSO presents a fluid-submerged spring-damper-mass system. It would be susceptible to accelerating forces from locomotion, such as the vertical oscillations during flying and running. Under the influence of limb and body accelerations, the LSO soft tissues could physically oscillate, moving within the CSF. These oscillations could present multiple potential physical mechanisms for mechanosensation. First, Necker proposed the hypothesis that CSF flow through the TGs could elicit a mechanosensing response in the accessory lobes, based on analogy to the vestibular system in the mammalian inner ear (Necker 1999, 2005; Necker 2006). However, the accessory lobes do not possess the stereocilia typical of hair cells of the inner ear, which are specialized for the detection of fluid motion (Schroeder and Murray 1987). Recently identified intraspinal mechanosensors in zebrafish do possess microvilli or cilia that extend into the CSF, consistent with the potential for fluid sensing (Wyart 2009; Hubbard et al. 2016; Henderson et al. 2019).

Furthermore, fluid-based mechanosensing does not fully explain the novel anatomical features of the denticulate ligaments and glycogen body of the LSO. Alternatively, work by Schroeder suggests a strain mechanosensing hypothesis. Schroeder’s investigation of the ultra-structural features of the avian accessory lobes revealed a close association between the accessory lobes and denticulate ligaments and features consistent with strain or pressure mechanosensors similar to peripheral proprioceptors (Schroeder and Murray 1987). Based on the morphology and biomechanical properties of the LSO, we suggest that the anatomy is more consistent with strain-based mechanosensing, producing accelerometer-like function. However, it is also possible that the LSO achieves both gyroscopic and acceleration sensing through a combination of multiple types of mechanosensing neurons in the accessory lobes, with some responding to fluctuations in fluid flow or pressure and some responding to strain in the denticulate ligaments. Specifically, the accessory lobes are found on the left and right ventro-lateral margins of the spinal cord (Necker 1997). Their pair-wise, segmental configuration opens the possibility for differential sensing, where rolling motions could be sensed as the difference in sensor activation between left and right, and pitching motion as the difference between proximal and distal pairs.

Future directions: If the LSO acts like an inertial measurement sensor, this presents the potential for morphological diversity in the LSO structure among birds, associated with different locomotor ecologies. Variations in the properties of the bony canal space, ligaments network and glycogen body could vary the directional sensitivity and frequency response characteristics of the LSO structures, acting to tune the LSO to specific locomotor demands. For example, a larger or higher density glycogen body would increase the sensitivity of the LSO to external accelerations. Variation in the denticulate ligament network morphology could tune the directional sensitivity and frequency response characteristics. Variations in the canal space or TGs’ prominence could enhance fluid flow, which could play a direct role in mechanosensing, as suggested by Necker (Necker 2006), or tune the magnitudes of soft tissue oscillations by enabling fluid flow around the moving soft tissues.

Once a physical model of LSO mechanosensing has been developed, this would enable model-based predictions of morphological variations related to different locomotor ecologies. For example, swimmers and divers experience relatively low vertical accelerations, have few visual cues underwater, and might therefore require more sensitive angular sensing compared to translational sensing. Tree climbers must accurately balance in 3D—suggesting that they should have small glycogen body mass and thin ligaments to enable rapid, high-frequency responses to small angular fluctuations. Specialized runners experience high leg impact forces, which should require a robust network of ligaments to limit the excursions of the spinal cord, with an accentuated fluid space to act as a fluid damper. In addition to the LSO structure specialization between species, we might also expect changes through ontogeny to tune balance sensing to the changing locomotor capabilities and demands on juvenile body structures.

## Conclusion

We have generated a 3D reconstruction of the adult common quail’s lumbosacral spinal canal, including the soft tissue and the denticulate ligament network. Our morphological data are based on the combination of digital dissection of a contrast-enhanced uCT scan and classical dissection of smaller soft tissue structures of the denticulate ligaments network below the spinal cord. Our morphometric analysis suggests an updated hypothesis for LSO mechanosensing, in which the combined structure of glycogen body, spinal cord, and denticulate ligaments could be tuned to oscillate in response to locomotor accelerations. The TGs may act as CSF reliefs to enable fluid flow around moving soft tissues. The combined LSO morphology is a fluid-submerged spring-damper-mass system that suggests an accelerometer-like balance sensing function.
